# Effect of non-invasive rhythm control on outcomes in patients with first diagnosed atrial fibrillation presenting to an emergency department

**DOI:** 10.1186/s12873-025-01194-z

**Published:** 2025-03-03

**Authors:** Christian Salbach, Mustafa Yildirim, Hauke Hund, Matthias Müller-Hennessen, Norbert Frey, Hugo Anton Katus, Evangelos Giannitsis, Barbara Ruth Milles

**Affiliations:** Department of Internal Medicine III, Cardiology, University Hospital of Heidelberg, Im Neuenheimer Feld 410, 69120 Heidelberg, Germany

**Keywords:** Atrial fibrillation, Real-world evidence, First diagnosed atrial fibrillation, Non-invasive rhythm control

## Abstract

**Background:**

Evidence suggests a benefit of a rhythm control approach in patients with a recent diagnosis of atrial fibrillation (AF). This study sought to evaluate clinical characteristics, treatment strategies and outcomes in patients with first diagnosed AF (FDAF) undergoing a non-invasive rhythm control strategy in an emergency department (ED).

**Methods:**

This analysis uses data from the Heidelberg Registry of Atrial Fibrillation (HERA-FIB). HERA-FIB is a retrospective single-centre observational study which consecutively included patients presenting to the ED of the University Hospital of Heidelberg between June 2009 and March 2020 with a sequential follow-up for all-cause mortality, stroke, major bleeding events and myocardial infarction (MI). Outcomes of patients with FDAF were related to treatment strategy (non-invasive rhythm vs. rate control).

**Results:**

Among the 2,758 (27%) patients who presented with FDAF, a non-implementation of a non-invasive rhythm control strategy at admission was observed in 75.4% and associated with an excess of all-cause mortality hazard ratio (HR): 1.61 (95%CI 1.30–1.99), *p* < 0.0001 and incident MI HR: 1.88 (95% CI 1.22–2.90), *p* = 0.0043 during follow-up. The non-implementation of a non-invasive rhythm control remained an independent predictor for all-cause mortality and MI even after adjustment for significant univariate variables with an adjusted HR of 1.52 (95%CI: 1.14–2.04, *p* = 0.0043) and 1.89 (95%CI: 1.03–3.45, *p* = 0.0392), respectively.

**Conclusion:**

Real-world data from FDAF patients presenting to an ED showed a benefit regarding all-cause mortality and MI favouring a non-invasive rhythm control strategy. Further prospective research is needed to validate this hypothesis.

**Trial registration:**

The trial was registered at ClinicalTrials.gov Identifier: NCT05995561.

**Supplementary Information:**

The online version contains supplementary material available at 10.1186/s12873-025-01194-z.

## Background

Following the diagnosis of atrial fibrillation (AF), key elements in management of the disease include stroke prevention, symptom control and cardiovascular risk factor management [1, 2]. To alleviate symptoms, current guidelines not only advocate for rate- but also for rhythm control. However, there is no specific recommendation regarding the long term effects of rhythm vs. rate control on major cardiovascular outcomes (MACE) [1]. In recent years there here has been evolving evidence pointing towards a paradigm shift for the management of patients with first diagnosed atrial fibrillation (FDAF). The EAST AFNET 4 (Early Treatment of Atrial Fibrillation for Stroke Prevention Trial) trial revealed that irrespective of symptoms, an early rhythm control approach including ablation and cardioversion resulted in a reduction of the composite endpoint consisting of cardiovascular death, stroke and hospitalization due to heart failure and acute coronary syndrome [3]. Mainly attributed to the development and implementation of ablation procedures for these patients, evidence suggests a benefit of rhythm control and ablation in patients with FDAF [4]. However, the aforementioned research relies on carefully chosen outpatient cohorts, with thorough control over confounding factors and relatively short follow-up periods and are not suitable for the setting of an emergency department (ED). Therefore, this study aims to assess real-world evidence for prevalence, risk factors and outcomes of an acute non-invasive rhythm control approach in an unselected cohort of FDAF patients presenting to an ED.

## Methods

### Study population, study design and follow-up

**Fig. 1:**
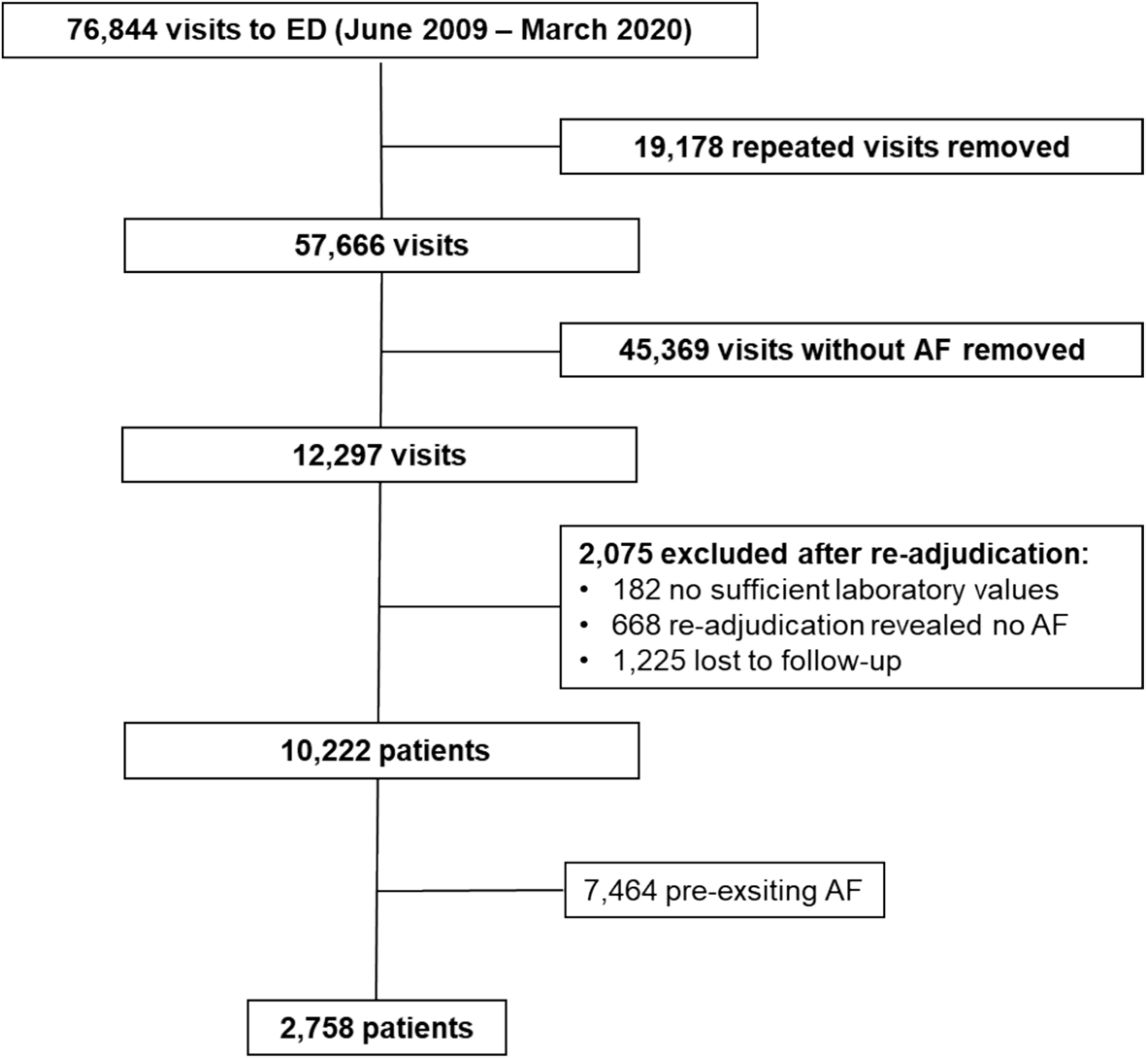
Flow chart of in- and excluded patients. The diagram shows included and excluded patients after removing repeated visits, visits without AF, re-adjudication of the diagnosis, adjudication of follow-up and exclusion of patients with pre-existing AF. Abbreviations: AF, atrial fibrillation; ED, emergency department

The study population of this retrospective observational single-centre study consisted of patients included in the Heidelberg Registry of Atrial Fibrillation (HERA-FIB). HERA-FIB consecutively enrolled AF patients admitted to the ED of the department of cardiology of the Heidelberg University Hospital from June 2009 to March 2020. The study population, inclusion and exclusion criteria and aims of HERA-FIB have been published earlier [5]. Inclusion criteria were comprehensive including age ≥ 18 years and AF either as primary reason for presentation or as a comorbidity. Exclusion criteria were a non-availability of a highly sensitive cardiac troponin T (hs-cTnT) value or a lost to follow-up for all-cause mortality. Within the HERA-FIB a sequential follow-up method was used to ensure high follow-up rates. This included the review of internal data and additional structured telephone calls, questionnaires and consultation of data from registration offices [5]. Within this study, the HERA-FIB cohort was stratified by first-time diagnosed AF (FDAF) or pre-existing AF. For this study patients with pre-existing AF were excluded (Fig. 1). This retrospective observational study had no influence on patient treatment. All treatments were provided at the discretion of the treating physicians. This study was conducted according to ethical principles stated in the Declaration of Helsinki (2008). This study was approved by the Ethics Committee of the University of Heidelberg (S-377/2013). Since this study involves no patients and uses data from clinical routine care, a consent to participate declaration was not applicable. HERA-FIB is registered at ClinicalTrials.gov. (NCT05995561).

### Data availability

The datasets analysed during the current study are not publicly available due to privacy concerns, but are available from the corresponding author upon reasonable request.

### Definitions

In line with the current guideline of the European Society of Cardiology (ESC), FDAF was defined as previously undiagnosed and undocumented AF regardless of symptom status and duration of AF episode [1]. A non-invasive rhythm control strategy was defined as pharmacological or electrical cardioversion at index event. Ablations for AF are not part of the routine care in the ED as they do not qualify as emergency treatment. Patients receiving a primary ablation strategy (i.e. urgent referral for ablation) were not assigned to the non-invasive rhythm control group. Initiation of a permanent oral anti-arrhythmic treatment is rare in the ED setting and was also not included in our definition of a non-invasive rhythm control strategy, as the effects may be delayed and uptake of the recommendation is not documented. If an electrical and pharmacological cardioversion was performed within the same patient, they were assigned to the electrical cardioversion group. Left ventricular ejection fraction (LVEF) was categorized according to the guidelines recommended by the American Society of Echocardiography (ASE) and the European Association of Cardiovascular Imaging (EACVI) [6]. The anticoagulation protocol during emergency department rhythm control relied on standard operation procedure (SOP) implemented within the ED but remained at the discretion of the attending physician. SOPs for anticoagulation protocol strictly followed guidelines of the ESC in their latest version at the time of presentation. Briefly patients with a CHA2DS2-VASC Score of ≥ 2 in men and ≥ 3 in women recieved anticoagulation. Patients with a score below the cut off recieved anticoagulation, if a cardioversion was planned and for 4 weeks after cardioversion. Thereafter anticoagulation was decided by the treating physician.

Patients undergoing emergency PCI recieved appropriate antiplatelet treatment (aspirin, clopidogrel) other potent antiplatelet agents (prasugrel, ticagrelor) were only administered in the cathlab. In general OAC was administered as soon as possible.

Unless appropriate anticoagulation was documented or the onset of AF was unequivocally recent, a transesophageal echocardiogram for exclusion of intracardiac thrombus was performed prior to cardioversion. The only exception was emergency cardioversion which could not be delayed.

### Statistical analysis

Continuous variables were tested for normal distribution using the Kolmogorov–Smirnov test. Normally distributed data is presented as means (standard deviations, SD). Non-normally distributed data is presented as medians (25th, 75th percentiles, IQR). Kaplan–Meier estimates are shown as counts or percentages. Here, groups were compared with the log-rank test. For categorical variables groups were compared using chi-squared test or Fisher’s exact test. For continuous variables, unpaired Student’s t-test or Wilcoxon rank-sum test was used. A multivariate Cox proportional hazards regression was performed to determine predictors for outcome parameters. The proportional hazards assumption was tested using the Grambsch and Therneau method. A two tailed *P*-value of < 0.05 was considered to indicate statistical significance. Statistical analysis was performed using MedCalc (Version 20.105).

## Results

A total of 2,758 (27%) patients within the HERA-FIB cohort presented with FDAF, whereas 7,464 (73%) patients were presenting with pre-existing AF. Among FDAF patients presenting to the ED, a non-invasive rhythm control strategy was part of the disease management in 679 (24.6%) patients, compared to 2,079 (75.4%) patients which did not receive a rhythm control approach. A rhythm control approach consisted of 576 (84.8%) electrical- and 103 (15.2%) pharmacological cardioversions. Baseline characteristics for FDAF patients are reported classified by patients with or without a non-invasive rhythm control strategy (Table 1). The groups differ significantly regarding baseline characteristics. Patients assigned to a rhythm control strategy were younger and had fewer co-morbidities including prior cardiovascular events, history of myocardial infarction (MI), previous coronary artery disease, transient ischemic attack or stroke (Table 1). The median heart rate at presentation was higher in patients with a non-invasive rhythm control approach. Biomarkers such as C-reactive protein and hs-cTnT concentrations were lower in AF patients with a non-invasive rhythm control approach. A comparison of available baseline characteristics for in- and excluded patients within HERA-FIB is shown in the supplement (Table S1).

**Table 1:** Baseline characteristics for FDAF patients classified by rhythm control strategy within the ED

Variables	Rhythm control *n* = 679	No-rhythm control *n* = 2079	*p*-value
Age, median (IQR)	69 (58–78)	73 (64–81)	< 0.0001
Sex, male, (n_%all_)	399 (58.8)	110 (53.4)	0.0146
HF, bpm, median (IQR)	129 (108–145)	108 (84–132)	< 0.0001
BMI, kg/m^2^, median (IQR)	27.5 (24.4–32.6) *n* = 514	26.8 (24.1–30.7) *n* = 1,135	0.0097
Hs-cTnT ng/L, median (IQR)	15 (9–26)	16 (9–35)	0.0233
CRP, mg/L, median (IQR)	4.7 (2.0–13.7) *n* = 677	5.7 (2.0–21.2) *n* = 2,072	< 0.0001
Creatinine, mg/dL, median (IQR)	0.94 (0.79–1.14)	0.93 (0.76–1.15)	0.5025
eGFR (CKF-EPI) ml/min median (IQR)	77 (59–91)	74 (54–89)	0.0174
NTproBNP, ng/L, median (IQR)	3,370 (1396–6472) *n* = 298	2,482 (772–7302) *n* = 800	0.0307
Arterial Hypertension, (n_%all_)	493 (72.6)	1,587 (76.3)	0.0502
Diabetes mellitus, (n_%all_)	86 (12.7)	325 (15.6)	0.0595
Prior CABG, (n_%all_)	28 (4.1)	126 (6.1)	0.0564
Prior MI, (n_%all_)	65 (9.6)	281 (13.5)	0.0071
Prior PAD, (n_%all_)	35 (5.2)	127 (6.1)	0.3587
Prior CAD, (n_%all_)	172 (25.3)	661 (31.8)	0.0015
Prior TIA/stroke, (n_%all_)	56 (8.2)	193 (9.3)	< 0.0001
Prior malignancy, (n_%all_)	91 (14.4)	418 (20.1)	0.0001
Prior COPD, (n_%all_)	48 (7.1)	172 (8.3)	0.3148
Left ventricular Ejection fraction	*n* = 582	*n* = 1,119	
Normal LVEF	282 (48.5)	610 (54.5)	0.0176
Mild abnormal LVEF	105 (18.0)	220 (19.7)	0.4204
Moderately abnormal LVEF	115 (19.8)	168 (15.0)	0.0127
Severely abnormal LVEF	80 (13.7)	121 (10.8)	0.0756
Oral anticoagulation, (n_%all_)	522 (76.9)	1284 (61.8)	< 0.0001
VKA, (n_%all_)	110 (21.1)	366 (28.5)	0.0012
DOAC, (n_%all_)	412 (78.9)	918 (71.5)	0.0012
Antiarrhythmic drugs*, (n_%all_)	65 (9.8)	21 (1.0)	< 0.0001
ß-blockers, (n_%all_)	544 (80.1)	1,600 (77.0)	0.0860
ACE/AT-1 inhibitors, (n_%all_)	464 (68.3)	1215 (58.4)	< 0.0001
Ca-channel blockers, (n_%all_)	157 (23.1)	485 (23.3)	0.9121
Digitalis, (n_%all_)	55 (8.5)	201 (9.7)	0.2216

^*^antiarrhythmic drugs included amiodarone, mexiletine, dronedarone, sotalol, propafenone or flecainide

Abbreviations: *ACE* Angiotensin Converting Enzyme, *AF* Atrial fibrillation, *BMI* Body mass index, *bpm* beats per minutes, *bp* blood pressure, *CRP* C-reactive protein, *CAD* Coronary artery disease, *CABG* Coronary artery bypass graft, *dia* diastolic, *COPD* Chronic obstructive pulmonary disease, *eGFR* estimated glomerular filtration rate according to CKD EPI formula, *FDAF* First diagnosed atrial fibrillation, *HF* Heart frequency, *hs-cTnT* high sensitive cardiac troponin T, *IQR* Interquartile range, *LVEF* Left ventricular ejection fraction, *MI* Myocardial infarction, *DOAC* Direct oral anticoagulant, *NTproBNP* N-terminal-pro brain natriuretic peptide, *PAD* Peripheral artery disease, *sys* systolic, *TIA* Transient ischemic attack, *VAK* Vitamin K antagonist

### Outcomes stratified by rhythm control strategy

**Fig. 2:**
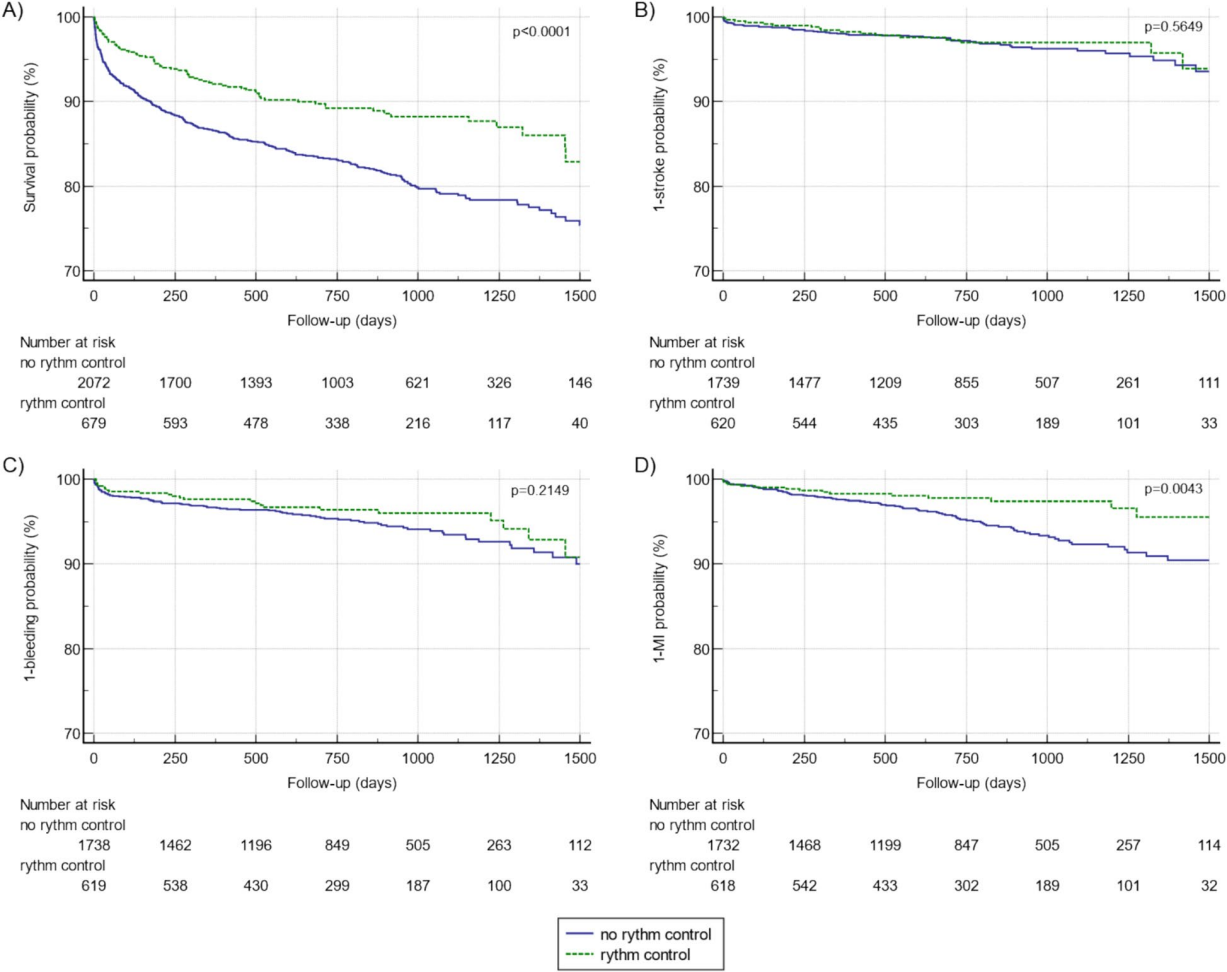
Kaplan Meier analysis for cardiovascular events in FDAF patients stratified by non-invasive rhythm control strategy for all-cause mortality (**A**), stroke (**B**), major bleeding events (**C**) and myocardial infarction (**D**). Abbreviations: FDAF, first diagnosed atrial fibrillation; MI, myocardial infarction

Rates for all-cause mortality and MI showed a significant difference in patients with FDAF and non-invasive rhythm control vs. no-rhythm control strategy (Table 2). During a median follow-up of 24 months (IQR 13–35) patients without a rhythm control strategy showed a HR of 1.61 (95%CI 1.30–1.99, *p* < 0.0001) for all-cause mortality and a HR of 1.88 (95%CI 1.22–2.90, *p* = 0.0043) for incident MI (Fig. 2). Temporal relationship between acute rhythm control and adverse outcomes are shown in supplementary Table 2. Effects of rhythm control on all-cause mortality reflect a prompter relation to the intervention, whereas effects on MI reflect long term effects of the management strategy. A breakdown for causes of mortality (cardiac vs. non-cardiac) revealed higher mortality rates for both cardiac and non-cardiac death in patients without a non-invasive rhythm control approach showing a HR for cardiac death of 1.59 (95%CI: 1.07–2.37), *p* = 0.0235 and a HR for non-cardiac death of 1.62 (95%CI: 1.23–2.08), *p* = 0.0002.

**Table 2:** Outcomes of first diagnosed AF patients stratified by rhythm control strategy

Variables	Non-invasive rhythm control *n* = 679		No-rhythm control *n* = 2079	*p*-value
All-cause mortality, n(%_all_)	74 (10.4)	378 (18.2)		< 0.0001
HR (95%CI)		1.61 (1.30–1.99)		< 0.0001
Cardiac-death*, n(%_all_)	21 (3.1)	106 (5.1)		0.0304
HR (95%CI)		1.59 (1.07–2.37)		0.0235
Non-cardiac death, n(%_all_)	53 (7.8)	272 (13.1)		0.0002
HR (95%CI)		1.62 (1.23–2.08)		0.0002
Stroke, n(%_all_)	17 (2.7)	55 (3.1)		0.6158
HR (95%CI)		1.17 (0.69–1.97)		0.5649
Major bleeding, n(%_all_)	25 (4.0)	92 (5.3)		0.2252
HR (95%CI)		1.30 (0.86–1.96)		0.2149
Myocardial infarction, n(%_all_)	15 (2.4)	90 (5.2)		0.0045
HR (95%CI)		1.88 (1.22–2.90)		0.0043

Abbreviations: *defined as death related to an acute of chronic cardiac disease such as myocardial infarction, arrhythmia, chronic heart failure with decompensation. HR, hazard ratio; CI, confidence interval

Even after adjustment for age, sex, presence of diabetes mellitus, prior peripheral- or coronary artery disease, prior MI, prior cancer, left-ventricular ejection fraction, kidney function and c-reactive protein, a non-implementation of a rhythm control in FDAF patients remained an independent predictor for all-cause mortality with an adjusted HR (aHR) of 1.52 (95%CI: 1.14–2.04), *p* = 0.0043. Other independent predictors for all-cause mortality in FDAF patients were age, diabetes mellitus, prior cancer, moderate and severely abnormal LVEF, eGRF < 60 ml/min and c-reactive protein > 5 mg/L. (Table 3). Adjusted survival curves are shown in supplementary Fig. 1. Additionally, the non-implementation of a rhythm control strategy remained an independent predictor of an incident MI aHR: 1.89 (1.03–3.45), *p* = 0.0392, other independent associated variables with incident MI were diabetes mellitus and eGFR < 60 mL/min (Table 4).

**Table 3:** Cox-proportional HR model for all-cause mortality in patients with FDAF

Covariate	aHR (95%CI)	*p*-value
**Age, per year**	**1.06 (1.04–1.07)**	** < 0.0001**
Sex, female	0.92 (0.73–1.21)	0.5357
**Diabetes mellitus**	**1.68 (1.29–2.20)**	**0.0001**
Prior PAD	1.40 (0.97–2.02)	0.0713
Prior CAD	1.04 (0.77–1.40)	0.8172
Prior MI	1.04 (0.73–1.48)	0.8264
**Prior cancer**	**1.39 (1.04–1.86)**	**0.0244**
Mildly abnormal LVEF	1.16 (0.83–1.63)	0.3819
**Moderately abnormal LVEF**	**1.70 (1.23–2.35)**	**0.0013**
**Severely abnormal LVEF**	**2.64 (1.90–3.67)**	** < 0.0001**
**eGFR < 60 mL/min**	**1.75 (1.35–2.27)**	** < 0,0001**
**CRP > 5 mg/L**	**1.89 (1.43–2.51)**	** < 0.0001**
**No-rhythm control**	**1.52 (1.14–2.04)**	**0.0043**

Abbreviations: *aHR* adjusted Hazard ratio, *CAD* Coronary artery disease, *CI* Confidence interval, *CRP* C-reactive protein, *eGFR* estimated glomerular filtration rate, *LVEF* Left ventricular ejection fraction, *MI* Myocardial infarction, *PAD* Peripheral artery disease

**Table 4:** Cox-proportional HR model for incident MI in FDAF patients

Covariate	aHR (95%CI)	*p*-value
Age, per year	1.01 (0.99–1.04)	0.2013
Sex, female	1.16 (0.70–1.94)	0.5647
**Diabetes mellitus**	**2.50 (1.49–4.18)**	**0.0005**
Prior PAD	0.64 (0.23–1.80)	0.3994
Prior CAD	1.44 (0.79–2.64)	0.2367
Prior MI	1.82 (0.95–3.50)	0.0727
Prior cancer	1.13 (0.64–2.01)	0.6758
Mildly abnormal LVEF	1.72 (0.94–3.15)	0.0767
Moderately abnormal LVEF	1.51 (0.78–2.91)	0.2200
Severely abnormal LVEF	1.01 (0.43–2.36)	0.9902
**eGFR < 60 mL/min**	**2.26 (1.33–3.82)**	**0.0025**
CRP > 5 mg/L	1.47 (0.64–2.01)	0.1638
**No-rhythm control**	**1.89 (1.03–3.45)**	**0.0392**

Abbreviations: *aHR* adjusted Hazard ratio, *CAD* Coronary artery disease, *CI* Confidence interval, *CRP* C-reactive protein, *eGFR* estimated glomerular filtration rate, *LVEF* Left ventricular ejection fraction, *MI* Myocardial infarction, *PAD* Peripheral artery disease

### Differences in outcomes of rhythm control strategies

We observed higher rates for all-cause mortality within patients recieving a pharmacological cardioversion, compared to patients assigned to electrical cardioversion as primary rhythm control strategy. HR for all-cause mortality in patients receiving a pharmacological cardioversion was 3.61 (95%CI 1.85–7.04), *p* = 0.0002 (Table 5). Even after adjustment for age, sex, diabetes mellitus, prior peripheral- and coronary artery disease, MI and prior cancer, as well as LVEF, eGFR < 60 ml/min and c-reactive protein, pharmacological cardioversion remained an independent predictor for all-cause mortality showing an aHR of 2.51 (95%CI: 1.38–4.58), *p* = 0.0026 (Table 6).

**Table 5:** Outcomes and hazard ratios for FDAF patients stratified by electrical vs. pharmacological cardioversion

Variables	Electrical CV *n* = 576	Pharmacological CV *n* = 103	*p*-value
All-cause mortality, n(%_all_)	53 (9.2)	21 (20.4)	0.0008
HR (95%CI)		3.61 (1.85–7.04)	0.0002
Cardiac-death*, n(%_all_)	13 (2.3)	8 (7.8)	< 0.0001
HR (95%CI)		8.03 (2.3–27.6)	0.0001
Non-cardiac death, n(%_all_)	40 (6.9)	13 (12.6)	< 0.0001
HR (95%CI)		2.62 (1.19–5.78)	0.0172
Stroke, n(%_all_)	13 (2.5)	4 (4.3)	0.3183
HR (95%CI)		2.38 (0.59–9.58)	0.2231
Major bleeding, n(%_all_)	24 (4.6)	1 (1.1)	0.1156
HR (95%CI)		0.45 (0.14–1.46)	0.1824
Myocardial infarction, n(%_all_)	12 (2.3)	3 (3.2)	0.5854
HR (95%CI)		1.70 (0.39–7.42)	0.4785

Abbreviations: *HR* Hazard ratio, *CI* Confidence interval, *CV* Cardioversion

^*^defined as death related to an acute of chronic cardiac disease such as myocardial infarction, arrhythmia, chronic heart failure with decompensation

**Table 6:** Cox-proportional HR model for incident all-cause mortality in FDAF patients stratified by electrical vs. pharmacological cardioversion

Covariate	aHR (95%CI)	*p*-value
**Age, per year**	**1.05 (1.02–1.08)**	**0.0011**
Sex, female	0.84 (0.48–1.47)	0.5436
Diabetes mellitus	1.02 (0.54–1.96)	0.9325
**Prior PAD**	**2.12 (1.01–4.45)**	**0.0476**
Prior CAD	1.22 (0.63–2.35)	0.5562
Prior MI	1.10 (0.48–2.53)	0.8215
Prior cancer	0.58 (0.23–1.47)	0.2466
Mildly abnormal LVEF	0.94 (0.42–2.11)	0.9380
Moderately abnormal LVEF	1.49 (0.74–2.99)	0.2613
**Severely abnormal LVEF**	**2.23 (1.09–4.57)**	**0.0284**
**eGFR < 60 mL/min**	**2.46 (1.41–4.29)**	**0.0015**
**CRP > 5 mg/L**	**2.52 (1.40–4.53)**	**0.0021**
**Pharmacological CV**	**2.51 (1.38–4.58)**	**0.0026**

Abbreviations: *aHR* adjusted Hazard ratio, *CAD* Coronary artery disease, *CV* Cardioversion, *CI* Confidence interval, *CRP* C-reactive protein, *eGFR* estimated glomerular filtration rate, *LVEF* Left ventricular ejection fraction, *MI* Myocardial infarction, *PAD* Peripheral artery disease

## Discussion

This study reports several noteworthy findings on the utilization and outcomes of non-invasive rhythm control in patients with first diagnosed AF presenting in an ED setting. Using real-world data from 10,222 AF patients presenting consecutively over 11-years, we demonstrated that 27% of all AF patients were FDAF patients. Among these, non-invasive management strategies encompassing electrical or pharmacological cardioversion were only sparsely utilized in 24.6%. Additionally, we could show that patients not treated with a non-invasive rhythm control strategy show higher HRs for all-cause mortality (HR 1.61; 95% CI 1.30–1.99) and incident MI (HR 1.88; 95% CI 1.22–2.90) during follow-up.

There is conflicting evidence regarding the benefit of an early rhythm control approach in unselected AF patients and a lack of evidence for the adequate initial treatment strategy for FDAF patients presenting in an ED setting. Given, that AF is the most common supraventricular tachycardia encountered in the ED and FDAF is a frequent reason for a presentation, there is an imperative need to enhance management and outcomes of AF patients presenting to an ED [7].

Recent studies including CABANBA, EAST-AFNET or CASTLE-AF are focusing on the evaluation of a benefit for a rhythm control approach utilizing an ablation strategy in an outpatient cohort of AF patients [3, 8, 9]. However, in the acute clinical setting of an ED a primary ablation strategy is often neither available nor feasible.

In our cohort, patients recieving a non-invasive rhythm control approach were significantly younger than those who did not (69 vs. 73 years, *p* < 0.0001). However, the age of patients undergoing rhythm control aligns with data from the RE-LY registry, which reports a median age of 69.4 years (IQR 62–78) for Western Europe [10]. In FDAF patients, those undergoing cardioversion were more often male (58.8% vs. 53.4%, *p* = 0.0146). This aligns with the higher prevalence of AF in men [11, 12]. Previous studies have shown that women presenting to the ED with AF are on average older, show more comorbidities and suffer a higher symptomatic burden of the disease [13]. Therefore, the difference in rhythm control strategies may be explained by a difference in the clinical risk profile of male and female patients, not the symptomatic burden itself. FDAF patients undergoing a rhythm control approach exhibited significantly higher heart rates (129 vs. 108 bpm, *p* < 0.001) compared to those receiving a conservative treatment. It has previously been described that symptomatic burden of AF correlates with higher heart rates [14]. Thus, our finding of higher heart rates in the non-invasive rhythm control group might reflect the ESC guideline recommendations of utilizing a rhythm control approach as a tool for symptom relief [1, 2]. Patients receiving a cardioversion had an overall beneficial cardiac risk profile with fewer histories of MI, coronary artery disease and TIA or stroke. These findings are in line with previous work on risk factors precipitating the development of AF [1, 15–18]. In particular the low rates of stroke in FDAF patients are not unexpected, as studies report up to 25% of patients with an ischemic stroke or TIA are first diagnosed with AF during the neurological workup which often includes an intensified screening for AF [19]. These finding are also in line with previous work suggesting that AF may be triggered by cardiac remodelling including vascular dysfunction, underlining the important role of risk factor and comorbidity management in AF patients [20, 21]. Within HERA-FIB, there was a clear preference for electrical cardioversions (20.9% vs. 3.7%). This preference may be attributed to local experience, as well as on established evidence [22], since guidelines prefer an electrical-over pharmacological cardioversion for accelerated effects [23, 24].

In our study, we observed an excess in all-cause mortality in FDAF patients recieving a conservative treatment. This finding is in line with current results from a large metanalyses [25, 26] which included the EAST–AFNET 4 study [27], as well as the AFFIRM study [28] and other retrospective observational studies [29–33]. However, our finding contrasts with previous data from large registries, which indicated no survival benefit of early cardioversion [34, 35]. Albeit, these studies focused on patients with a long history of AF and are therefore not comparable to our cohort of FDAF patients receiving a non-invasive rhythm control approach in an ED setting.

The observed beneficial effect of rhythm control on survival may be attributed to several factors. Cardioversion may improve left ventricular ejection fraction, particularly in tachymyopathies, since arrhythmia progression is associated with adverse outcomes [36]. Notably, patients undergoing rhythm control strategies showed a beneficial baseline risk profile in in particular in regards to history of coronary artery disease and MI which may contribute to the lower all-cause mortality during follow-up. Previous studies have highlighted the fact that patients undergoing an early rhythm control tend to have a more favourable risk profile [28, 30, 37]. Proirettie et al. demonstrated that the favourable effect of an early rhythm control vanishes in a fully adjusted Cox-Regression model for patients from the ESC EORP-AF General Long-Term Registry [30]. In this cohort, even after adjustment for other risk factors, rhythm control during the index event remained an independent predictor for all-cause mortality. It is noteworthy that in our regression analysis a history of coronary artery disease and MI were not relevant risk factors for all-cause mortality. Within our analysis independent predictors were age, diabetes mellitus, history of cancer, moderately to severely reduced LVEF, eGFR < 60 mL/min and CRP > 5 mg/l. Additionally, we observed that FDAF patients undergoing an initial non-invasive rhythm control approach suffered fewer incident MIs during follow-up. The EAST-AFNET 4 trial had a combined endpoint which included hospitalization for MI, however MI was not addressed by itself [27]. On the other hand AF during MI has been associated with poor outcomes [38]. A Swedish observational study showed an increased risk of MI recurrence in 90 day follow-up with any kind of AF during the index MI [39]. The observed lower MI rates for FDAF patients receiving a non-invasive rhythm control approach represents a new aspect of AF management.

In our study, we report diabetes mellitus, eGFR < 60 mL/min and a non-invasive rhythm control strategy to be independent predictors for incident MI. Thus, as previously discussed we also observed significant differences in baseline characteristics including comorbidities such as prior of coronary artery disease and age, which might also affect the result upon the follow-up. This might suggest that patients not recieving a rhythm control have more comorbidities and thus represent a cohort of patients with higher frailty than those recieving rhythm control, putting them at higher risk for MI. However, diabetes mellitus was not significantly different at baseline which suggest an added effect to the baseline differences. Despite the difference in baseline charcterisitics and independent effect of rhythm control in MI could be explained by improved left ventricular ejection fraction in sinus rhythm [40] and reduced arrhythmia progression [41]. Furthermore longer diastolic filling times after rhythm control could improve cardiac perfusion [42] and reduce oxidative stress which is implicated in the pathogenesis of AF as well as ischemic heart disease [43, 44].

Notably, we did not observe an impact of a non-invasive rhythm control approach on incident stroke events. This demonstrates appropriate exclusion of intracardiac thrombus material prior to a non-invasive-rhythm control approach. Overall our real-world data showed lower rates of strokes compared to other studies including the EAST-AFNET trial [27, 28]. A rhythm control approach has not clearly been proven to be beneficial for stroke risk reduction on its own. [27, 28, 45, 46] The expert consensus on atrial cardiomyopathies has endorsed the concept of endocardial remodelling [20]. Cardiac remodelling is related to pro thrombogenic structural changes in relation to AF which are independent of the current heart rhythm, suggesting little effect of rhythm control on stroke risk [47]. In contrast a recent study investigating further stroke events in patients with FDAF during the index stroke showed a reduced stroke recurrence at 12 months, if a rhythm control was performed in under 2 months after the index event [31]. These results however are not comparable to our current study cohort as AF cohorts are vastly different. In our study, the implementation of a non-invasive rhythm control approach had no impact on major bleeding events, which is in line with current evidence suggesting an appropriate anticoagulation in both groups [25, 28, 29, 33].

In an exploratory analysis, we observed that patients who recieved a pharmacological cardioversion showed worse survival rates compared to those recieving electrical cardioversion. A recent metanalysis showed no difference in the efficacy of cardioversion between pharmacological and electrical cardioversion in FDAF patients in the ED [48]. Studies included within this metanalysis often preferred an electrical cardioversion, if the primary pharmacological cardioversion failed. In our analysis patients which recieved an electrical cardioversion in addition to a pharmacological cardioversion were assigned to the electrical cardioversion group. A lower conversion rate with pharmacological cardioversion alone as compared to electrical cardioversion has been described, which may also explain the unfavourable outcomes we observed [49, 50].

Additionally, it could be hypothesized that pharmacological cardioversion was mainly applied to patients with an adverse risk profile given the higher risk for the conscious sedation required for electrical cardioversion [49]. However, further analysis is required to verify this theory.

### Strengths

The results of our study derived from a single centre real-world registry consecutively including patients with AF who presented to an ED without substantial exclusion criteria. This inclusive study design offers unique insights, which differ from the evidence generated from major RCT and other registries of AF patients. Many recent RCTs exclude patients with recurrent or persistent AF, advanced renal failure, women of childbearing age and patients with triggered AF episodes [3, 51]. Additionally, elderly or patients with chronic kidney dysfunction or patients with prosthetic valves and mitral stenosis tend to be excluded from RCTs leaving a gap in evidence for these patient populations [3, 52–55]. The period of observation of our study spans from 2009 to 2020 and is therefore largely unaffected by the latest findings of the EAST-AFNET 4 [3]. While some studies are limited to symptomatic patients our analysis included all patients with AF. Additionally, we report a long follow-up period for the manifestation of cardiovascular events.

### Limitations

The current analysis is based on an observational single-centre study on AF patients in the clinical setting of an ED. Therefore, several limitations have to be considered. The study design and setting reduces the generalizability of our results. Data acquisition was retrospective which might lead to limited availability of some data and human errors leading to incorrect or incomplete data which could not fully be ruled out. Additionally, we did not systematically collect follow-up data on time in sinus rhythm, prohibiting a meaningful analysis concerning this outcome. Finally, despite all efforts, a selection bias due to the exclusion of patients lost to follow-up could not be entirely ruled out. Overall the exclusion rates in this consecutive registry were low given the fact that 76,844 visits by 57,666 patients were screened. The vast majority of cases (45,369) had to be excluded because patients did not meet inclusion criteria since they did not have AF. Compared to this number 1,225 cases lost to follow-up are very few cases. It should also be pointed out that patients requiring intensive care were not seen in the ED but transferred straight to the ICU and low risk patients were treated by an on call general physician, leading to an exclusion of very sick and very healthy patients. Additionally, the impact of rhythm control on MI might be confounded by the comorbidities and frailer patient collective, which was not assigned to a rhythm control strategy.

## Conclusions

The implementation of a non-invasive rhythm control approach in AF patients presenting to an ED is not only a powerful tool to reduce AF burden but was associated with a reduction in all-cause mortality and MI in patients with FDAF. However, further prospective studies are needed to confirm this hypothesis.

## Supplementary Information

Supplementary Material 1

## Data Availability

The datasets analysed in ths study is not publicly available due to privacy regulations and ethical concerns.

## Abbreviations

AF: Atrial fibrillation
aHR: Adjusted hazard ratio
CABANA: Catheter Ablation vs. Antiarrhythmic Drug Therapy for Atrial Fibrillation
CASTLE-AF: Catheter Ablation versus Standard Conventional Therapy in Patients with Left Ventricular Dysfunction and Atrial Fibrillation
EAST AFNET 4: Early Treatment of Atrial Fibrillation for Stroke Prevention Trial
ED: Emergency department
EHRA: European Heart Rhythm Association
ESC: European Society of Cardiology
FDAF: First diagnosed atrial fibrillation
HERA-FIB: Heidelberg Registry of Atrial Fibrillation
HR: Hazard ratio
MACE: Major cardiovascular event
MI: Myocardial infarction
DOAC: Direct oral anticoagulants
OAC: Oral anticoagulants
VKA: Vitamin-K antagonists
