# Supplementary material for: Effect of non-invasive rhythm control on outcomes in patients with first diagnosed atrial fibrillation presenting to an emergency department
Source: BMC Emerg Med. 2025 Mar 3;25:35. doi: 10.1186/s12873-025-01194-z (PMC11877945; doi:10.1186/s12873-025-01194-z)
Supplement: Supplementary file 1 — Supplementary Material 1 [file 12873_2025_1194_MOESM1_ESM.docx]

**Supplementary Materials:**

**Supplementary tables:**

**Table S1. Comparison of baseline characteristics between in- and excluded patients.**

| **Variables** | **Included patients**  **n=2,758** | **Excluded patients**  **n=9,539** | **p-value** |
| --- | --- | --- | --- |
| Age, median (IQR) | 73 (62-80) | 75 (66-82) | <0.0001 |
| Sex, male, (n_%all_) | 1,509 (54.7) | 5,703 (59.8) | <0.0001 |
| HF, bpm, median (IQR) | 113 (88-136) | 84 (70-106) | <0.0001 |
|  |  | n=9,516 |  |
| BMI, kg/m^2^, median (IQR) | 27.12 (24.2-31.1)  n=1,649 | 26.5 (23.9-30.4)  n=5,873 | 0.0067 |
| Hs-cTnT ng/L, median (IQR) | 16 (9-31) | 19 (10-37) | <0.0001 |
| CRP, mg/L, median (IQR) | 5.3 (2.0-18.6)  n=2,749 | 6.3 (2.0-20.7)  n=9,365 | 0.0017 |
| Creatinine, mg/dL, median (IQR) | 0.93 (0.77-1.15) | 1.00 (0.81-1.31) | <0.0001 |
| eGFR, ml/min, median (IQR) | 75.3 (56.3-89.7) | 67.1 (46.3-84.7) | <0.0001 |
| NTproBNP, ng/L, median (IQR) | 2,770 (908-6,952)  n=1,098 | 3,003 (1,089-7,916)  n=4,105 | 0.0251 |
| Arterial Hypertension, (n_%all_) | 2,080 (75.4) | 7,872 (82.5) | <0.0001 |
| Diabetes mellitus, (n_%all_) | 411 (14.9) | 1,987 (20.8) | <0.0001 |
| Prior CABG, (n_%all_) | 154 (5.6) | 909 (9.5) | <0.0001 |
| Prior MI, (n_%all_) | 346 (12.5) | 1,533 (16.1) | 0.0071 |
| Prior PAD, (n_%all_) | 162 (5.9) | 720 (7.5) | 0.0027 |
| Prior CAD, (n_%all_) | 833 (30.2) | 4,102 (43.0) | <0.0001 |
| Prior TIA/stroke, (n_%all_) | 249 (9.0) | 1,192 (12.5) | <0.0001 |

Abbreviations: AF, atrial fibrillation; BMI, body mass index; bpm, beats per minutes; bp, blood pressure; CRP, C-reactive protein; CAD, coronary artery disease; CABG, coronary artery bypass graft; dia, diastolic; FDAF, first diagnosed atrial fibrillation; HF heart frequency; hs-cTnT, high sensitive cardiac troponin T; IQR, interquartile range; MI, myocardial infarction; NTproBNP, n-terminal-pro brain natriuretic peptide; PAD, peripheral artery disease; sys, systolic; TIA, transient ischemic attack.

**Supplementary table 2.** Mean follow-up (days) on outcome parameters.

| **Variables** | **Positive outcomes**  **follow-up (days), mean (IQR)** |
| --- | --- |
| **All-cause mortality** | **145 (26-424)** |
| Rhythm control | 194 (45-470) |
| No-rhythm control | 136 (24-418) |
| **Stroke** | **294 (35-703)** |
| Rhythm control | 302 (113-582) |
| No-rhythm control | 278 (32-748) |
| **Major bleeding** | **243 (24-723)** |
| Rhythm control | 265 (34-743) |
| No-rhythm control | 211 (22-733) |
| **Myocardial infarction** | **454 (156-797)** |
| Rhythm control | 236 (24-605) |
| No-rhythm control | 487 (170-801) |

Abbreviations: IQR, interquartile range.

*
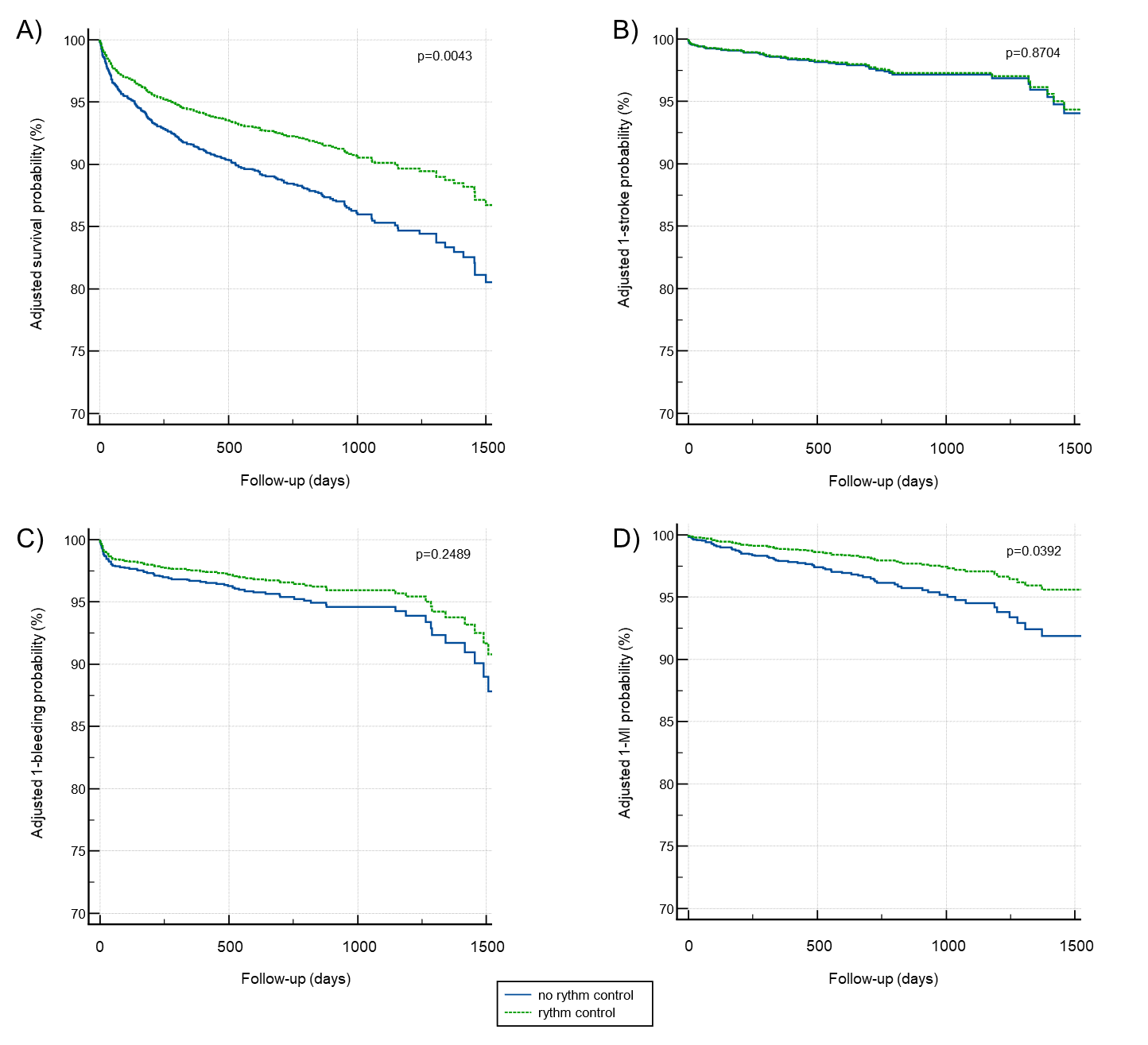
*

**Supplementary figure 1. Adjusted survival curves using cox-regression model for all-cause mortality (A), stroke (B) major bleeding (C) and MI (D).** Abbreviation: MI, myocardial infarction.
